# Insights from a novel monogenic autoinflammatory disease: overview of a multicentric European cohort of 38 patients with COPA syndrome

**DOI:** 10.1016/j.ard.2025.09.013

**Published:** 2025-10-25

**Authors:** Clémence David, Nadia Nathan, Eslam Al-Abadi, Peter D. Arkwright, Brigitte Bader-Meunier, Sebastian Becker, Alexandre Belot, Mary Brennan, Sylvain Breton, Vincent Bondet, Jacques Cadranel, Aurore Coulomb l'Hermine, Sébastien De Almeida, Darragh Duffy, Teresa Clavaguera Poch, Alix de Becdelièvre, Siham El Khalifi-Boulisfane, Marco Gattorno, Montse Gispert-Saüch, Florian Gothe, Yves Hatchuel, Daniela Herdliczko, Ayse Ayzit Kilinc, Vaclav Koucky, Géraldine Labouret, Raffaele Manna, Hélène Maillard, María Luisa Matoses Ruipérez, Caterina Matucci-Cerinic, Anna Mensa-Vilaro, Katarzyna Michel, Thierry Jo Molina, Berta Lopez Montesinos, William G. Newman, Julia Papenkort, Christina Rapp, Cinthia Rames, Gillian I Rice, Markus A. Rose, Heloise Reumaux, Laurent Sailler, Nicolaus Schwerk, Luis Seabra, Jérémie Sellam, Andrea Taddio, Caroline Thumerelle, Alberto Tommasini, Maud Tusseau, Stefano Volpi, Martin Wetzke, Laurence Weiss, Anne Welfringer-Morin, Marie Wislez, Matthias Griese, Yanick J Crow, Marie-Louise Frémond

**Affiliations:** 1 Laboratory of Neurogenetics and Neuroinflammation, Imagine Institute, Paris, France; 2 Department of Internal Medicine, Hôpital Bichat-Claude Bernard, Assistance Publique Hôpitaux de Paris, Université Paris Cité, Paris, France; 3 Paediatric Pulmonology Department and Reference Centre for Rare Lung Diseases RespiRare, INSERM UMR_S933 Laboratory of Childhood Genetic Diseases, Armand Trousseau Hospital, Sorbonne University and APHP, 75012 Paris, France; 4 Childhood Arthritis and Rheumatic Diseases Unit, Birmingham Women’s and Children’s Hospital NHSFT, Birmingham, United Kingdom; 5 Lydia Becker Institute of Immunology and Inflammation, University of Manchester, Manchester, United Kingdom; 6 Department of Paediatric Haematology-Immunology and Rheumatology, Necker-Enfants Malades Hospital, Assistance Publique - Hôpitaux de Paris (APHP), and Reference Centre for Inflammatory Rheumatism, Autoimmune Diseases and Systemic Interferonopathies in Children (RAISE), Université Paris Cité, Paris, France; 7 Pneumology Department, Darmstädter Kinderkliniken Prinzessin Margaret, Darmstadt, Germany; 8 Member of the chILD-EU register group; 9 National Reference Center for Inflammatory Rheumatism, Autoimmune Diseases and Systemic Interferonopathies (RAISE), Paediatric Nephrology, Rheumatology, Dermatology Unit, Hospital of Mother and Child, Hospices Civils of Lyon, Lyon, France; 10 Paediatric & Adolescent Rheumatology, Royal Hospital for Children & Young People, Edinburgh, United Kingdom; 11 Pediatric Radiology Department, Hôpital Necker-Enfants Malades, AP-HP Centre Université de Paris, Paris, France; 12 Translational Immunology Unit, Institut Pasteur, Université Paris Cité, Paris, France; 13 Department of Pneumology and Thoracic Oncology, Tenon Hospital, Assistance Publique-Hôpitaux de Paris (AP-HP) Sorbonne Université, Paris, France; 14 Pathology Department, Trousseau University Hospital, Assistance Publique-Hôpitaux de Paris, Sorbonne Université, Paris, France; 15 Department of Internal Medicine, CHU de Toulouse, Toulouse, France; 16 UF Paediatric Rheumatology Department, Hospital Doctor Trueta, Girona, Spain; 17 Laboratory of Genetics, Henri Mondor University Hospital, APHP, University Paris Est Creteil, INSERM, IMRB, Creteil, France; 18 Emergency, Infectious Disease and Paediatric Rheumatology Department, Centre Hospitalier Régional Universitaire Lille, University of Lille, Lille, France; 19 UOC Reumatologia e Malattie Autoinfiammatore, IRCCS Istituto Giannina Gaslini, Genova, Italy; 20 Pediatric Pneumology, Dr von Hauner Children´s Hospital, University of Munich, German Center for Lung Research (DZL), Munich, Germany; 21 Department of General Paediatrics, Competence Centre for Inflammatory Rheumatism, Autoimmune Diseases and Systemic Interferonopathies (RAISE) Antilles-Guyane, EpiCliV Research Unit, University of the French West Indies, Martinique University Hospital, Fort-de France, France; 22 Rheumatology, Universitäts - Kinderspital Zürich, Zurich, Switzerland; 23 Istanbul University-Cerrahpasa, Cerrahpasa Faculty of Medicine, Division of Pediatric Pulmonology, Istanbul, Turkey; 24 Department of Paediatrics, 2nd Faculty of Medicine, Charles University and Motol University Hospital, Prague, Czech Republic; 25 Paediatric Pulmonology Department, University Hospital for Children, Toulouse, France; 26 Department of Internal Medicine and Clinical Immunology, Referral Centre for Rare Systemic Autoimmune Diseases in the North of France, Northwest, Mediterranean and Guadeloupe (CeRAINOM), CHU de Lille, 59000 Lille, France; 27 Section of Pediatric Nephrology, Hospital Universitari i Politècnic (HUIP) La Fe, Valencia, Spain; 28 Department of Neurosciences, Rehabilitation, Ophthalmology, Genetics, Maternal and Child Health (DINOGMI), University of Genoa, Italy; 29 Department of Immunology-CDB, Hospital Clínic-IDIBAPS, Barcelona, Spain; 30 Pathology Department, Hôpital Necker-Enfants Malades, Assistance Publique-Hôpitaux de Paris, Centre- Université de Paris, Paris, France; 31 Section of Pediatric Rheumatology, Hospital Universitari i Politècnic (HUIP) La Fe, Valencia, Spain; 32 Manchester Centre for Genomic Medicine, Manchester University NHS Foundation Trust, Manchester, United Kingdom; 33 Division of Evolution Infection & Genomic Sciences, School of Biological Sciences, Faculty of Biology, Medicine and Health, University of Manchester, Manchester, United Kingdom; 34 Stuttgart Children’s Hospital. Center for Congenital Lung Diseases, Stuttgart, Germany; 35 Paediatric Pneumology and Allergology Unit, CHU of Amiens, Amiens, France; 36 Paediatric Rheumatology Unit, University of Lille, Jeanne de Flandre Hospital, Lille, France; 37 Department of Paediatrics, German Center for Lung Research, Medical University of Hannover, Hannover, Germany; 38 Department of Rheumatology, Saint-Antoine Hospital, APHP, Centre de Recherche Saint-Antoine (CRSA) Inserm UMRS_938, Sorbonne Université, 184 Rue de Faubourg Saint-Antoine, 75012 Paris, France; 39 Department of Pediatrics, Institute for Maternal and Child Health IRCCS “Burlo Garofolo”, Trieste, Italy; 40 Department of Medical, Surgical and Health Sciences University of Trieste Trieste Italy; 41 Paediatric Pulmonology and Allergy Department, Hôpital Jeanne de Flandre, CHU Lille, Univ. Lille, Lille, France; 42 Centre International de Recherche en Infectiologie, Inserm, U1111, University Claude Bernard, Lyon 1, UMR5308, ENS de Lyon, Lyon, France; 43 Hospices Civils de Lyon, Department of Medical Genetics, University Hospital of Lyon, Lyon, France; 44 Pediatric Pulmonology, Strasbourg University Hospital, France; 45 Department of Dermatology, Reference Centre for Genodermatoses and Rare Skin Disease (MAGEC), Necker-Enfants Malades Hospital, Assistance Publique-Hôpitaux de Paris, Université Paris Cité, Paris, France; 46 Service de Pneumologie, Hôpital Cochin, AP-HP, Inserm U1138, Université Paris Cité, Paris, France; 47 MRC Human Genetics Unit, Institute of Genetics and Cancer, University of Edinburgh, Edinburgh, United Kingdom

## Abstract

**Objectives:**

COPA syndrome is a rare monogenic autoinflammatory disease due to heterozygous mutations in *COPA*. It has phenotypic overlap with SAVI (STING-associated vasculopathy with onset in infancy), although the spectrum of clinical manifestations is not yet fully defined. Our aim was to better delineate the clinical phenotype of this rare disorder in a European cohort.

**Methods:**

Assessment of clinical, imaging and immunological data from 46 individuals (29 families) carrying a *COPA* mutation.

**Results:**

Among the 46 individuals carrying a *COPA* mutation, thirty-eight had at least one clinical manifestation likely related to their mutant-state (clinical penetrance of 83%). Twenty-two (58%) symptomatic patients were female, with a median age at disease onset of 3 years (range 0-50 years). Pulmonary involvement was observed in 34 patients, with interstitial lung disease in most cases (n=31) and diffuse alveolar haemorrhage in 11 individuals. Twenty-six patients demonstrated joint involvement, and seven had documented kidney disease. Previously undescribed features included skin (n = 12), cardiac (n = 8), gastrointestinal (n = 7) and hepatic involvement (n = 5). All but one patient tested positive for autoantibodies, and increased interferon signalling was noted in all those tested. Twenty-two patients were treated with JAK inhibitors with promising efficacy.

**Conclusion:**

We report a large European cohort of patients with COPA syndrome. While confirming the core organ features (lung, joint and kidney) of the disease, our data expand the phenotype to include cardiac, skin and gastrointestinal features, further demonstrating the clinical overlap with SAVI and other type I interferonopathies.

## Introduction

COPA syndrome is a monogenic autoinflammatory disease originally described in 2015 due to heterozygous mutations in the gene *COPA*, encoding the coatomer subunit α [1]. In the original paper, five families were reported, with symptomatic individuals variably manifesting interstitial lung disease (ILD), inflammatory arthritis and high autoantibody titres [2]. The authors hypothesised that the disease resulted from defective retrograde transport between the Golgi and endoplasmic reticulum (ER) mediated by the coat protein complex I (COPI) complex. A major advance in the understanding of COPA syndrome pathogenesis came from the observation of an enhanced expression of interferon (IFN)-stimulated genes (ISGs) in the whole blood of symptomatic individuals, a hallmark of the type I interferonopathies [3]. In addition, clinical overlap was noted with the well-defined type I interferonopathy due to gain-of-function (GOF) mutations in *STING1* [4], termed STING-associated vasculopathy with onset in infancy (SAVI). STING activation was subsequently shown to be a key driver of the pathogenesis of COPA syndrome [5–8]. Indeed, dominant-negative mutations in COPA prevent STING retrograde transport back to the ER from the Golgi, with associated chronic activation of STING and the induction of the expression of type 1 IFNs and Nuclear Factor kappa-B (NF-ĸB)-related inflammatory cytokines [5–9].

Since COPA syndrome was first described approximately 70 cases have been published [2,3,6,10–19]. The core clinical features are lung inflammation with either recurrent alveolar haemorrhage (AH) or ILD [20], joint disease that may mimic juvenile idiopathic arthritis [10,11,21], and renal involvement [12] with mainly glomerular disease. Given the pivotal role of type I IFN overproduction in the pathogenesis of COPA syndrome, JAK inhibition has been proposed as a targeted therapeutic strategy, with apparently promising effects in a number of case reports [13,21].

Here, we aimed to assemble a large European cohort of patients with molecularly confirmed COPA syndrome, thereby providing insights into the clinical and immunological spectrum of the disease which has been under-characterised due to the limited number of reported cases.

## Patients and Methods

### Patients

Patients with genetically confirmed COPA syndrome were included in this retrospective study. Thirteen patients have been previously reported [3,6,10,12,15,21,22]. Patients were recruited from referent rare diseases centres in France, Germany, Italy, Spain, Switzerland, Turkey, Czech Republic and the United Kingdom. Variants were annotated according to HGVS nomenclature using the MANE select transcript NM_004371.4. All patients had fulfilled the requirements of their local ethics committee for clinical data sharing.

### Data collection

Data collected included sex, clinical manifestations at presentation and during follow-up, and age at genetic diagnosis of COPA syndrome. Results of chest computed tomography (CT), pulmonary function tests (PFTs), bronchoalveolar lavage (BAL) fluid analyses and lung biopsies were recorded. ILD was defined according to CT scan findings, and AH either on CT scan and/or broncho-alveolar lavage (BAL) and/or lung biopsy. The presence of honeycombing and / or traction bronchiectasis and / or inter and intralobular septal thickening radiologically defined pulmonary fibrosis [23]. Biological parameters such as inflammatory markers, autoantibodies and immunological status were collected, as well as treatment characteristics and the response to therapy.

### IFN pathway assessment

Status of IFN biomarkers was determined by studying the expression of ISGs (by qPCR [24] or by NanoString [25]) in peripheral blood, and by measuring IFNα protein in serum using a Simoa ultra-sensitive digital ELISA [26].

### Study approval

The study was approved by the Comité de Protection des Personnes (ID-RCB/EUDRACT: 2014-A01017-40; revalidated in 2022). Written informed consent was obtained for all patients.

### Statistics

Data are expressed as median (range) or number (percentages). Analyses were performed using PRISM software (v10, GraphPad Inc.). A *p*-value less than 0.05 was considered significant.

## Results

### Demographics

Patients from 29 families with genetically confirmed COPA mutations were ascertained. Among these, 38 patients demonstrated at least one feature suggestive of COPA syndrome (Table 1 and Table S1), while 8 individuals were clinically asymptomatic after a full clinical evaluation, pulmonary function testing, lung CT imaging and assessment for proteinuria. These findings indicate a clinical penetrance of 83% where at least one individual in each family had been clinically ascertained. Twenty-two symptomatic patients were female (ratio F:M 1.4:1), while all asymptomatic COPA mutation carriers (n = 8), identified through family testing were female. Among symptomatic patients, the median age at disease onset was 3 (neonatal period-50) years, with 12 patients manifesting disease before the age of 5 years. The median age at molecular diagnosis was 13 (0.3-57) years. At last follow-up, 34 (89%) symptomatic patients were alive.

### Molecular data

**Figure 1:**
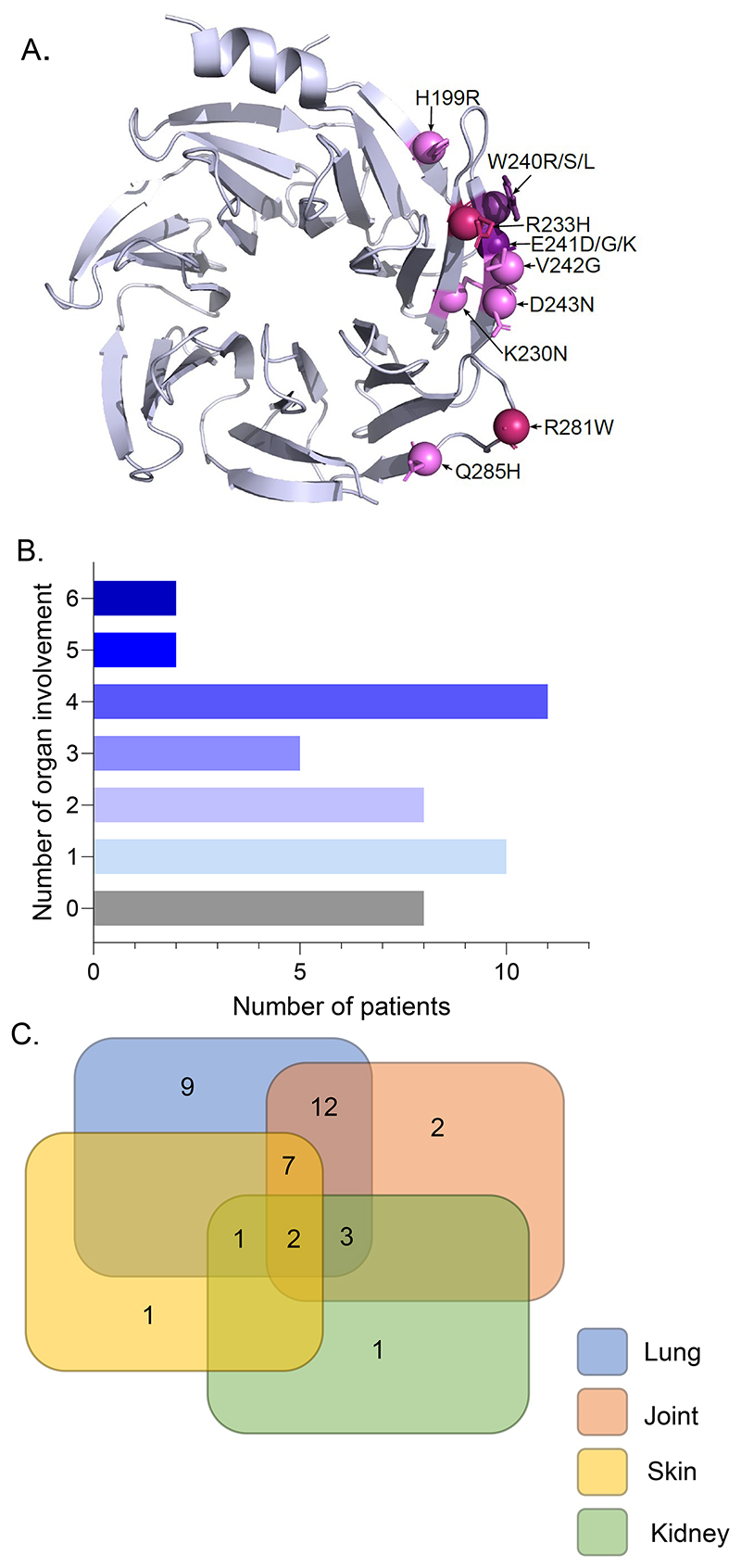
Clinical and molecular spectrum of COPA mutations. **A**. Location of pathogenic missense mutations within the crystal structure of COPA WD-repeat domain (PDB ID: 6PBG). Substitutions affecting W240 and E241 amino acids are shown in purple. The two most frequent substitutions reported in the cohort are represented in darker pink, with other mutations shown in light pink. **B**. Number of organs involved per patient in the cohort. **C**. Specific and overlapping features present in patients of the cohort, including lung, skin, joint and kidney disease.

All mutations were located in the well-defined mutational hotspot in the WD40 domain of the COPA protein (Figure 1A). Most mutations (61%) identified in symptomatic patients were inherited in a dominant manner from a mutation-positive parent, while 5 occurred *de novo* and 2 patients were somatic mosaic[27], with a variant allele frequency of 10% and 29% respectively. We were unable to test the parents of 8 patients (Table 2). Among symptomatic patients, the p.(Arg233His) (R233H) and p.(Arg281Trp) (R281W) substitutions were the most prevalent, occurring in 13 patients (34%) from 9 pedigrees and 9 patients (24%) from 5 pedigrees respectively. Additionally, 7 individuals carried a substitution of the amino acid p.(Trp240) (W240) (Table 2). Eight further substitutions were identified in single patients. Half of asymptomatic individuals (n = 4) carried the p.(Arg233His) (R233H) substitution (Table 2). No genotype-phenotype correlation was apparent, and the patients that were somatic mosaic for a *COPA* mutation did not present with a phenotype milder than those seen in the overall cohort (Table S1).

### Clinical features in symptomatic patients

Clinical characteristics of patients are summarised in Table 1 and detailed in Table S1. Features at onset are described in Table S1 where recorded. While 10 (26%) symptomatic patients manifest single organ involvement, most individuals experienced at least 2, and up to 6, organ involvement during the course of the disease (Figure 1B). Among patients with single organ involvement, pulmonary disease was the most common (n = 9) (Figure 1C). Regarding general features, 14 (37%) patients manifested failure to thrive and 9 (24%) recurrent fever.

#### Lung phenotype

**Figure 2:**
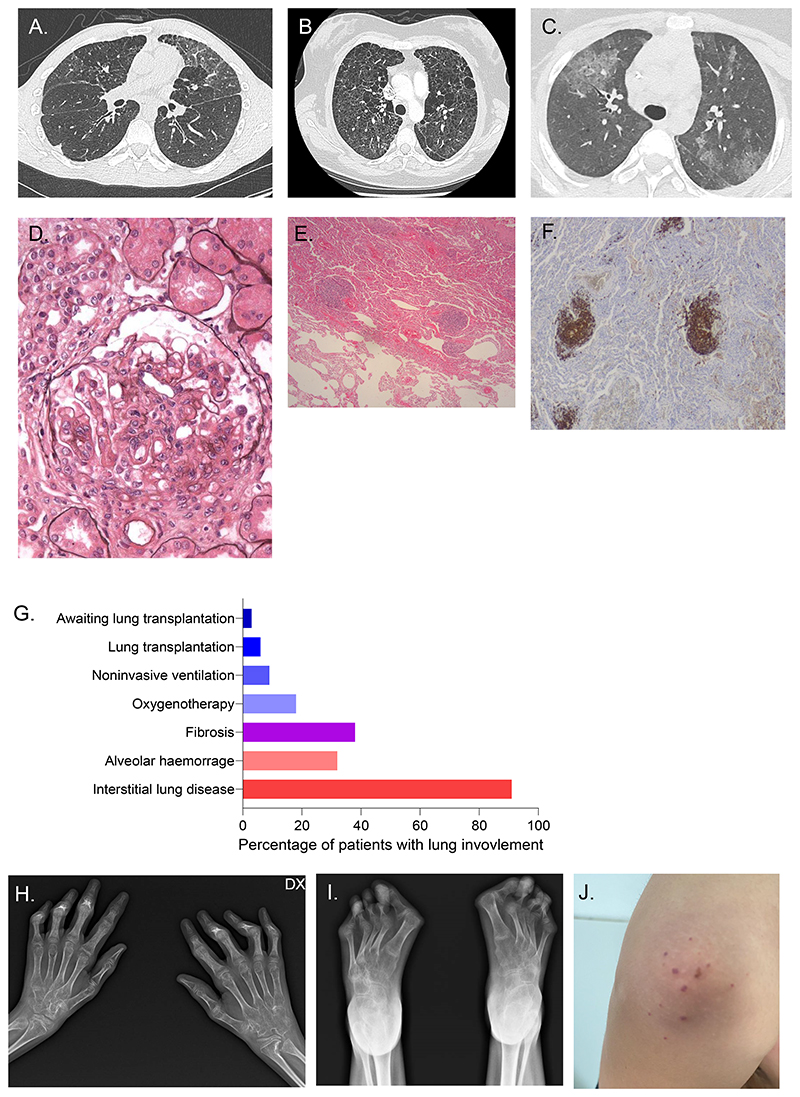
Representative lung imaging and joint disease in COPA syndrome. **A**. Chest CT scan of F3.P1 aged 27 years showing ground glass opacities, cysts and septal thickening. **B**. Chest CT scan of F5.P1 aged 24 years demonstrating diffuse cystic lesions. **C**. Chest CT scan of F1.P1 aged 12 years with recurrent AH. **D**. Kidney biopsy of F2.P4 showing wire-loop thickening of the capillary walls without endocapillary proliferation, presence of a hemi-circumferential cellular crescent and hypertrophy and vacuolation of podocytes. **E**. Haematoxylin and eosin (H&E) and Giemsa staining of lung biopsy of F6.P1 showing subpleural emphysema with local interstitial thickening of the remaining interalveolar septa and lymphoid follicles. **F**. CD20 staining of lymphoid follicles showing a predominance of CD20+ B cells. **G**. Distribution of pulmonary disease, and requirement for oxygen therapy, non-invasive ventilation or lung transplantation. **H**. Hand radiographs of F10.P1 aged 12 years showing diffuse demineralisation, bilateral carpal erosion and ankylosis, erosions with joint pinching of the right 2nd and 5th metacarpo-phalangeal spaces, and bilateral flaccid deformities of the proximal 2nd to 5th interphalangeal spaces. **I**. Foot radiographs of F10.P1 aged 12 years showing diffuse demineralisation and joint misalignment with bilateral major hallux valgus multifocal metatarsophalangeal dislocations. **J**. Purpuric lesions of the elbow of F26.P1 aged 13 years.

The lung was the most commonly affected organ in the cohort, with 34 (89%) symptomatic patients manifesting pulmonary disease. The majority of patients (n=21) complained of chronic cough and 14/23 (61%) of dyspnoea, while, at medical examination, 10 individuals had clubbing and tachypnoea, respectively. Only two patients reported episodes of haemoptysis (Table 3). The majority of patients were diagnosed with isolated ILD (n=23) (Table 3 and Figure 2G). Three patients had AH, and eight patients had both ILD and AH. Radiological (n=12) and/or histological (n=5) evidence of fibrosis was observed in 14 patients (41%), including patients aged less than 10 years. Of the 26 patients for whom a chest CT scan was available, ground glass opacities (n = 22) and cysts (n = 15) were the most frequently recorded anomalies, followed by honeycombing (n = 6), micronodules (n = 5), septal thickening (n = 10) and hilar lymphadenopathy (n = 5) (Figure 2A-C). Where PFTs were performed (n = 19), a restrictive pattern was seen in most patients (n = 13), with other patients displaying either an obstructive (n = 1) or mixed pattern (n = 4). BAL was performed in 16 patients, with fluid analysis revealing AH (n = 6), lymphocytic (n = 8) or neutrophilic alveolitis (n = 2). Lipid-laden macrophages were detected in 2 patients. Ten patients underwent lung biopsy, with follicular hyperplasia (i.e. the presence of non-clonal lymphocytic aggregates) observed in all cases (Figure 2E and 2F). In addition, inflammatory lymphocytic infiltrates were recorded in 6 patients, and signs of fibrosis in 5 individuals. Other histopathological findings included cholesterol pneumonitis, focal organizing pneumonitis, cellular non-specific interstitial pneumonia, fibrosing pleuritis and alveolar simplification. Six patients required long-term oxygen therapy, while disease in three patients necessitated non-invasive ventilation and one individual underwent invasive ventilation with extracorporeal membrane oxygenation during severe AH episodes. Two patients underwent lung transplantation at the age of 23 and 28 years respectively (See treatment section) and one other was considered as a candidate for lung transplant at the age of 30 years, but was not eligible due to other clinical disease features (Figure 2G).

#### Joint and muscle involvement

Joint involvement was frequent in the cohort (n = 26 patients, 68%). In particular, 17 (45%) patients presented with polyarthritis, often initially diagnosed as juvenile idiopathic polyarthritis, affecting small, large and axial joints. Of these, 4 patients suffered from severe disease with joint destruction (Figure 2H and 2I) and deforming arthritis, one of whom required bilateral knee replacement and now uses a wheelchair. Arthralgia without arthritis was seen in 6 patients. Of interest, two patients presented Jaccoud-like arthropathy as observed in other type I interferonopathies [28], and Boutonniere deformity of the fingers was documented in two further individuals.

#### Skin disease

Cutaneous manifestations were noted in 12 patients (32%), which were predominantly vascular. Notably, four patients presented with acral ulcers, chilblains or acrosyndrome, one with *livedo* and one with nasal perforation. Other skin features included purpura (n = 2) (Figure 2J), malar rash (n = 2), panniculitis (n = 1), oral ulcers (n = 1) and psoriasis (n = 1). Skin biopsy of a patient with purpuric lesions of the elbows showed focal leukocytoclastic vasculitis.

#### Kidney disease

Seven (18%) patients had renal disease. The most common histological pattern was pauci-immune glomerulonephritis associated with ANCA positivity (n = 3 with a specificity for anti-MPO). One patient had lupus-like glomerulonephritis with proliferative lesions associated with focal and segmental glomerulosclerosis and endomembranous deposits of C1q, C3, IgM, and IgG on immunofluorescence (Figure 2D). Another patient presented a lupus-like membranous glomerulonephritis with a full-house pattern on immunofluorescence and negative anti-phospholipase A2 receptor (PLA2R) antibodies. In addition, two patients had renal insufficiency without further clinical and histological characterisation. Two patients required kidney transplantation.

#### Other features

Five (13%) patients presented with hepatic involvement, including 4 individuals with chronic transaminitis, which occurred during methotrexate treatment in 2 cases. Liver biopsy was performed in 1 patient and showed no abnormalities. Seven (18%) patients presented gastrointestinal dysfunction i.e. gastro-oesophageal reflux disease (GERD) (n = 4), chronic diarrhoea (n = 2), gastrointestinal IgA vasculitis (n = 1) and stercoral peritonitis (n = 1). Cardiac involvement was identified in 8 (21%) patients. Pulmonary hypertension was found in 4 (11%) individuals, all presenting with ILD. Right heart catheterisation data were available for 2 patients, showing a pre-capillary pulmonary hypertension. Three patients were treated with phosphodiesterase 5 inhibitors combined with an endothelin receptor inhibitor in one case. The fourth patient affected by pulmonary hypertension had no specific treatment. Further cardiac features included myocarditis, mitral insufficiency, cardiac hypertrophy and unspecified heart failure (Table 1). Two patients had neurological involvement i.e. asymptomatic retro-cerebellar cyst in one patient and dyslexia with no brain imaging available in the second one. A patient with anti-neutrophil cytoplasmic antibody (ANCA) presented with nasal perforation and necrotising sinusitis. Finally, autoimmune thyroiditis was documented in one patient.

Asymptomatic carriers were all clinically assessed at molecular diagnosis (between the ages of 24 and 48 years) with normal respiratory examination. Pulmonary investigations (chest CT scan and PFTs) were performed in three carriers and were normal.

### Immunological features

#### Inflammatory markers

Inflammatory markers were available in less than half of the cohort. When tested, 6 (55%) patients had a dissociation of their inflammatory markers i.e. an elevated erythrocyte sedimentation rate (ESR) with normal levels of C-reactive protein (CRP) (Table S2). Specifically, 9/11 (82%) patients had a high ESR, while 12/19 (63%) and 7/19 (37%) patients had, respectively, normal or only mildly elevated CRP.

#### Type I IFN assessment

**Figure 3:**
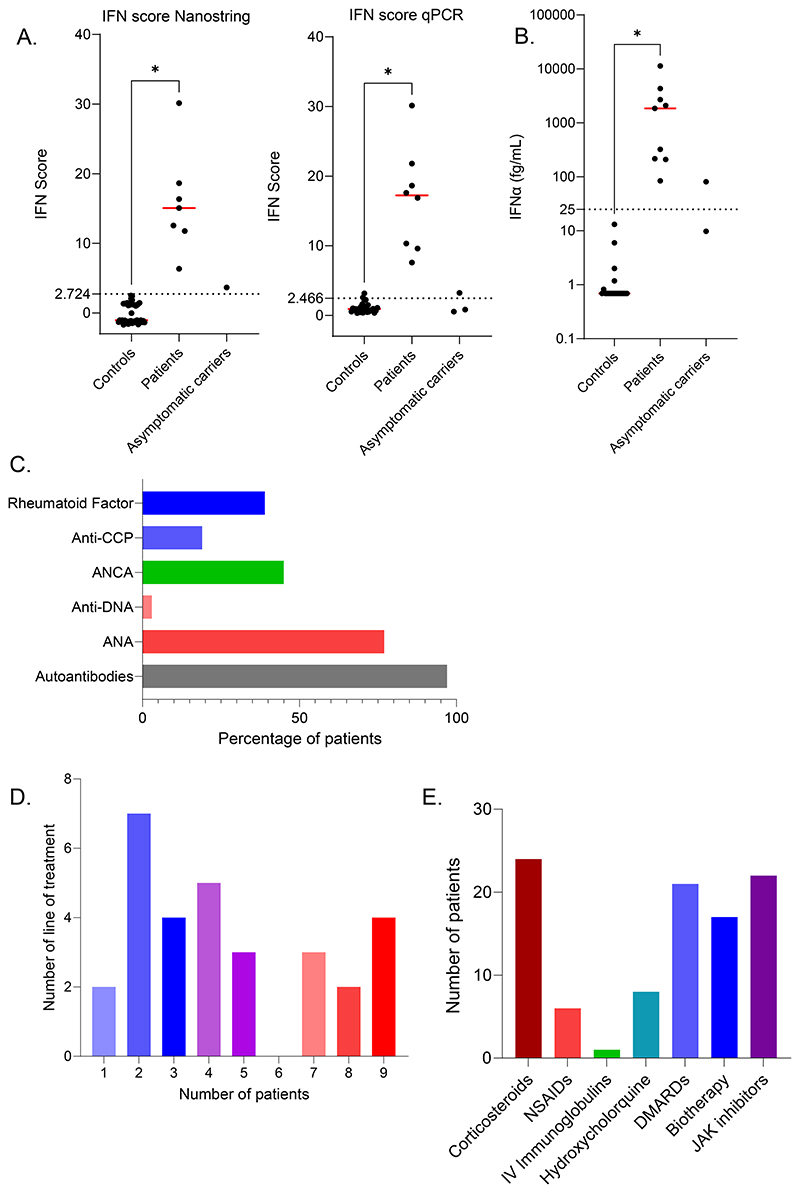
Immunological features and treatment exposures in symptomatic patients with COPA syndrome ascertained in this study. **A**. Interferon stimulated gene (ISG) expression measured in controls, patients and asymptomatic carriers, using either a 27 (left) or 6 (right) ISG panel (measured on a NanoString platform or using RT-qPCR respectively) to calculate an IFN score. **B**. Concentrations of IFNα protein assessed by ultra-sensitive digital ELISA (Simoa) in serum from healthy controls (n = 20), patients and asymptomatic carriers. **C**. Autoantibodies observed in the cohort: rheumatoid factor (RF), anti- citrullinated protein (CCP) antibodies, anti-neutrophil cytoplasm antibodies (ANCA), anti-double stranded DNA (anti-DNA), antinuclear antibodies (ANA). **D**. Number of lines of treatment received by each patient. **E**. Number of patients receiving corticosteroids, non-steroidal anti-inflammatory drugs (NSAIDs), intravenous (IV) immunoglobulins, hydroxychloroquine, disease-modifying antirheumatic drugs (DMARDs), biotherapy or JAK inhibitors. ****p <0.0001 using Mann-Whitney test.

All symptomatic patients evaluated (n = 20) had a positive type I IFN gene signature. Notably, half of the asymptomatic carriers tested also had a positive IFN signature (n = 2), albeit at a lower level than the symptomatic patients (Figure 3A). Serum IFNα protein levels measured by Simoa were high in all symptomatic patients tested (n = 9) (Figure 3B), and the 2 asymptomatic carriers had intermediate levels.

#### Autoantibodies and immunological features

When tested (n = 31), all but one symptomatic patient (97%) had positive autoantibodies (Figure 3C and Table S2). Twenty-four (77%) patients had positive antinuclear antibodies (ANA), almost all of which were nonspecific except for 5 patients (anti-DNA, n = 1, anti-RNP, n = 2, anti-centromere, n = 1, anti-Scl70, n = 1). Fourteen (45%) patients had positive ANCA antibodies (anti-MPO (n=9), anti-PR3 (n=2) or both anti-PR3 and anti-MPO (n=2)). Twelve (39%) patients were positive for rheumatoid factor (RF), and five (16%) had positive anti-cyclic citrullinated peptide (CCP) antibodies (Table S2). Six patients presented hypergammaglobulinemia.

Lymphocyte immunophenotyping was performed in 7 patients, showing variable modification of lymphocyte subpopulations.

### Therapeutics and outcome

#### Outcome

After a median follow-up period of 48 (6-96) months since the diagnosis of COPA syndrome, 34 patients were alive at last assessment with a median age of 14 (0.5-58) years. Four patients had died. Causes of death included fibrotic progression of ILD and suspected systemic lupus erythematosus without specification at the age of 35 years, respiratory failure at the age of 23 years, and cardiac arrest at home at the age of 31 years 4 years after lung transplantation. One patient died by suicide.

#### Treatment

The median number of different immunomodulatory treatments received by each patient was 4 (0-9). More than two-thirds of the patients received at least two immunomodulatory drugs (Table 4). Seventeen (45%) patients were treated intensively, receiving more than 4 different immune modulators (Figure 3D). Half (55%) of the patients received a disease-modifying antirheumatic drug (DMARD) or a biotherapy (47%) (Figure 3E) during the course of their disease. Twenty-two patients received a JAK inhibitor, most commonly baricitinib (n=17, 45%) (Table 4 and S3), with a median age at initiation of 13 (0.5-57) years and a median follow-up under treatment of 24 (0-72) months. Of these 22 patients, 16 (73%) had previously received a DMARD and/or a biotherapy without sufficient efficacy, thereby justifying the introduction of a JAK inhibitor. For sixteen patients, JAK inhibition was clinically assessed to have been associated with disease stabilisation or improvement: joint and lung disease improved in 7 patients each and stabilised in 1 and 6 patients respectively. However, despite treatment, disease progression was noted in 5 patients, including uncontrolled joint disease in 3 cases and lung disease progression in 2 patients. One additional patient had just started treatment. No serious infectious adverse events were noted except in one patient on high dose baricitinib who presented with meningococcal pneumonitis. Two patients received a lung transplantation and two patients received a kidney transplantation. The first patient who underwent lung transplantation had a favourable pulmonary outcome, but subsequently died of cardiac failure 4 years post-transplantation. The other one is stable 6 months post transplantation. Kidney function of the two patients who underwent kidney transplantation is stable 2 years post transplantation.

## Discussion

Previous case descriptions of COPA syndrome provided initial insights into the phenotypic pleiotropy of this rare autosomal dominant disorder [2,5–7,10,12,17,19,29]. The European cohort described here, comprising 38 symptomatic patients, confirms the known core features of COPA syndrome i.e. lung inflammation, arthritis and kidney disease [2], and further expands the disease spectrum.

In our cohort, most cases manifest early in life, although some cases demonstrated a later onset. Almost 90% of patients manifested pulmonary involvement. Some key features can be delineated to help clinicians diagnose COPA syndrome-related lung disease, which is of particular concern given the high associated morbidity due to progression to fibrosis and end-stage respiratory failure. Clinically, almost half of the patients had nail clubbing, as reported in other cases [14,17], which should be looked for in patients with unexplained autoinflammatory disease. However, lung disease can be strictly asymptomatic, therefore a chest CT scan and PFTs should be performed. As previously described [14], AH can be insidious – with only two patients in our cohort presenting with episodes of obvious haemoptysis, and must be looked for on chest imaging (X-ray or CT scan) or in BAL. Regarding lung CT imaging, the most common pathological pattern was of ground-glass opacities. In more than half of the patients, a particular pattern consisting of diffuse cystic lesions was observed. Such cystic lung lesions have also been reported in other genetic type I interferonopathies, most particularly SAVI [30,31]. These cystic lesions might be the radiological consequence of the follicular bronchiolitis or lymphoid follicles found in all patients on biopsy [32]. The lung CT abnormalities seen in our cohort were consistent with other case series [33], except for ‘crazy paving’ that we did not record. BAL analysis was not specific, with the observation of diverse histopathological patterns.

The second major organ involved was the musculoskeletal system, as previously reported [2,11,13,21], with a wide spectrum and severity of manifestations ranging from arthralgia to destructive polyarticular arthritis regardless of anti-CCP status. Although considered as a core feature of COPA syndrome [2,34], renal disease was recorded in less than a quarter of our cohort. The majority of patients presented with ANCA-like pauci-immune glomerulonephritis, highlighting the fact that COPA syndrome may mimic ANCA vasculitis, as also illustrated by the finding of nasal perforation and necrotising sinusitis in one patient. Given the high associated morbidity, renal disease should be searched for in all individuals carrying a pathogenic COPA mutation, with 2 patients in our cohort requiring kidney transplantation. Renal disease can be isolated, underscoring the need to enquire about family history and assess type I IFN signalling in patients with unexplained glomerulopathy [34].

We observed several clinical features not commonly, or previously, reported in COPA syndrome. Notably, almost one third of our cohort manifest skin disease, reminiscent in some cases of the skin vasculopathy seen in SAVI and other type I interferonopathies [35]. Other rare organ involvement included the hepatic, gastrointestinal and cardiac systems. While hepatic disease has only been previously reported once [29], we recorded 5 patients with hepatic manifestations in our cohort, highlighting the possibility of an underestimation of hepatic disease in COPA syndrome. Of note, two patients developed transaminitis after methotrexate, raising concerns about the use of hepatotoxic drugs in the context of COPA syndrome and other type I interferonopathies where severe liver damage has been observed in a few case reports [36,37]. Studies of metabolic dysfunction-associated steatotic liver disease and viral hepatitis suggest a deleterious effect of STING signalling on hepatocytes [38] that could account for a possible increased risk of hepatoxicity in monogenic diseases driven by STING activation. Twenty per cent of patients experienced gastrointestinal symptoms, primarily GERD, for which the direct involvement of COPA dysfunction is not clear. Since GERD can worsen lung inflammation, and has been reported in SAVI [30], it seems sensible to track and treat GERD in COPA patients [39]. Finally, pulmonary hypertension has never been described in the context of COPA syndrome. However, four patients in our cohort had World Health Organization (WHO) group 3 pulmonary hypertension. A direct effect of the type I IFN pathway in the development of pulmonary hypertension is also possible, as some evidence has been reported linking type I IFN and STING signalling to pulmonary hypertension [40,41] and pulmonary hypertension has been recorded in patients having type I interferonopathies without lung disease [42]. This study also highlighted another clinical overlap with SAVI: the lack of neurological involvement, which is a core feature of Aicardi-Goutières syndrome and other type I interferonopathies. The diversity of type I interferonopathy phenotypes is not fully understood but is likely to arise from differences in cellular or tissue sources of IFN. The number of patients with COPA syndrome reported in the literature is still small, and it is likely that the full extent of the associated clinical spectrum is yet to be defined. For these reasons, we have been comprehensive in our description of the clinical features observed in the patients described here. At the same time, we recognise that features such as vitiligo and GERD are seen at relatively high frequency in the general population, so that establishing a definitive relationship to COPA dysfunction is not possible at this time. Overall, the phenotypic spectrum of COPA syndrome is strikingly diverse and deserves careful clinical assessment, with some patients having isolated organ manifestation and others multi-organ involvement. As some patients develop novel organ involvement with age, physicians should continue to monitor patients over time.

COPA syndrome is an autosomal dominant disease due to heterozygous mutations inherited from a mutation-positive parent in most cases. In our cohort, the mutation occurred *de novo* in 4 patients. In addition, 2 patients presented with mosaicism, which has not been previously reported in COPA syndrome, but has been seen in other type I interferonopathies[43]. This observation indicates the requirement for a reduction of the variant allele frequency (VAF) detection threshold in molecular diagnosis. In addition to the possibility of *de novo* mutation or mosaicism, another challenge in the diagnosis of COPA syndrome is the high degree of clinical non-penetrance. Nearly 19% of the individuals in our cohort carried a pathogenic COPA mutation in the absence of clinical signs, a finding consistent with the literature [2]. All asymptomatic carriers in our cohort were women. However, several male asymptomatic carriers have been described by other teams [2]. Interestingly, half of the asymptomatic carriers presented a mildly elevated IFN score, suggesting that other factors - such as additional genetic, environmental, epigenetic influences [44] or monoallelic expression [45] – might play a protective or aggravating role in determining phenotypic status. When assessing the pathogenic prediction scores of *COPA* variants *in silico*, the predicted scores were high for all mutations except for the Q285H variant [10]. Of possible note, the Q285H variant was seen in a patient with a relatively mild phenotype, i.e. isolated joint disease. Except for this example, no genotype-phenotype correlation has been suggested or reported in COPA syndrome.

All of the mutations in our cohort were located in the same hotspot in the N-terminal part of COPA protein, encompassing exons 7, 8, 9 and 10. The location of these COPA mutations is directly linked to the pathogenesis of COPA syndrome as they affect the WD40 domain, which plays a key role in the recognition of STING for its retrograde transport. Recently, mutations in the C-terminal part of *COPA* have been published as causative of COPA syndrome [46]. These mutations do not seem to directly affect STING signalling and might be considered as causing a different disease with a more severe inflammatory phenotype. The precise pathogenesis of COPA syndrome is incompletely understood, as some features may be independent of IFN signalling [47] given that STING can mediate non-IFN related effects [47]. Moreover, *COPA* mutations may affect protein trafficking beyond STING [1], having been shown to (also) cause ER stress and upregulated Th17 immunity [2]. The use of novel therapies, specifically targeting type I IFN signalling, such as the monoclonal anti-IFNAR1 antibody anifrolumab [48], will help in discriminating the IFN-dependent and independent pathogenesis of COPA syndrome.

Symptomatic COPA patients experience a high disease burden, with the majority receiving multiple lines of immunosuppressive treatment. Although our study was not designed to assess the response to treatment, JAK inhibition targeting the type I interferon receptor downstream signalling was the most commonly used treatment approach, and was assessed to effectively control at least some aspect of the disease in more than two thirds of the cases - as previously reported by us and others [13,18,21]. Except for one patient who developed meningococcal pneumonia, no other serious adverse events were recorded in the cohort. However, as severe infectious events have been reported with JAK inhibition, all patients should be screened for VZV status and offered zoster vaccination when indicated, and BK, EBV and CMV viremia should be monitored for frequently during follow-up. Unfortunately, in some patients disease still progressed despite JAK inhibitor treatment, and end-stage organ failure occurred in several patients - as manifest by the requirement for lung and kidney transplantation in several patients in our cohort and in the literature [16,49]. Of note, lung transplantation in the context of COPA syndrome or SAVI is challenging, with poor outcomes in most patients [50,51]. Specifically, several patients experienced acute allograft dysfunction, with or without high levels of IL-6, highlighting the need to improve the immunosuppressant strategy in the context of constitutive activation of immune cells [16,49,50]. Understanding the specific and relative role of immune cells (monocytes, lymphocytes) and lung resident cells (alveolar epithelial cells, lung endothelial cells) in the pathogenesis of the lung inflammation seen in COPA syndrome and SAVI is crucial in order to optimise patient care [20].

Here, we describe, to our knowledge, the largest cohort of COPA patients yet reported. While confirming the known core phenotype of COPA syndrome, we have broadened the clinical spectrum of this severe type I interferonopathy to include other features. Pulmonary and renal disease drive morbidity and mortality in COPA syndrome and should be a focus to prevent progression to irreversible organ damage. The presence of *de novo* and mosaic mutations, in addition to clinical non-penetrance, should prompt clinicians to assess type I IFN signalling status and/or sequence *COPA* in the presence of a concordant phenotype, even in the absence of a family history. JAK inhibition is currently the most common therapeutic strategy used. In the future, the availability of novel treatments that specifically target the type I IFN pathway, particularly anifrolumab [48], will be worth considering in the treatment of COPA syndrome. In the meanwhile, disease pathogenesis remains incompletely understood, particularly the mechanism of lung inflammation [20], an understanding of which will require further functional and therapeutic studies to improve patient management.

## Supplementary Material

Supplementary Material

## Figures and Tables

**Table 1:** Clinical characteristics of affected patients with *COPA* pathogenic variants at last report

Demographics	n=38 patients
Sex ratio (F:M)	1.4:1
Age at onset	3 (0*-50)
Age at molecular diagnosis	13 (0.3-57)
Age at last report	15.5 (0.5-58)
Alive / dead at last report	34/4
**Organ involvement**	
Failure to thrive	14 (37%)
Recurrent fever	9 (24%)
Lung involvement	34 (89%)
*ILD*	31 (82%)
*AH*	3 (8%)
*ILD and AH*	8 (21%)
*Fibrosis*	14 (37%)
Joint disease	26 (68%)
*Polyarthritis*	17 (45%)
*Arthralgias*	6 (16%)
*Isolated Boutonniere deformity*	2 (5%)
*Joint disease without specification*	2 (5%)
Skin involvement	12 (32%)
*Purpura*	2 (6%)
*Malar rash*	2 (6%)
*Acral ulcers / acrosyndrome*	3 (8%)
*Livedo*	1 (3%)
*Oral ulcers*	1 (3%)
*Nasal perforation*	1 (3%)
*Vitiligo*	1 (3%)
*Panniculitis*	1 (3%)
*Psoriasis*	1 (3%)
*Chilblains*	1 (3%)
*Not specified skin disease*	1 (3%)
Kidney involvement	7 (18%)
*ANCA -associated glomerulonephritis*	3 (8%)
*Not specified kidney insufficiency*	2 (5%)
*Lupus-like glomerulonephritis*	1 (3%)
*Membranous glomerulonephritis*	1 (3%)
Liver involvement	5 (13%)
*Cytolytic hepatitis / secondary to* *methotrexate*	4 (11%) / 2 (5%)
*Hepatosplenomegaly*	1 (3%)
Gut involvement	7 (18%)
*Gastro-oesophageal reflux*	4 (11%)
*Chronic diarrhoea*	2 (5%)
*Digestive IgA vasculitis*	1 (3%)
*Stercoral peritonitis*	1 (3%)
Cardiac involvement	8 (21%)
*Pulmonary hypertension*	4 (11%)
*Myocarditis*	1 (3%)
*Mitral insufficiency*	1 (3%)
*Cardiac hypertrophy*	1 (3%)
*Not specified cardiac insufficiency*	1 (3%)
Ear-nose-throat involvement	1 (3%)
*Nasal perforation*	1 (3%)
*Necrotizing sinusitis*	1 (3%)
Other features	Retro-cerebellar cyst (n = 1), thyroiditis (n = 1), dyslexia (n = 1).

Abbreviations: AH: alveolar haemorrhage; ANCA: Anti-neutrophil cytoplasmic antibody; Ig: Immunoglobulin; ILD: Interstitial lung disease.

Data are presented as number (percentages) or median (range). Ages are given in years.

* Neonatal presentation.

**Table 2:** Clinical penetrance status of the COPA mutations identified in the cohort.

Mutation of *COPA*	Symptomatic patients (n=38)	Asymptomatic carriers (n=8)
**Amino acid substitution**		
Het: c.698G>A/p.(Arg233His), R233H	13 (34%)	4 (50%)
Het: c.841C>T/p.(Arg281Trp), R281W	9 (24%)	1 (12%)
Het: c.718T>C/p.(Trp240Arg), W240R	4 (11%)	1 (12%)
Het: c.719G>C/p.(Trp240Ser), W240S	2 (5%)	0
Het: c.719G>T/p.(Trp240Leu), W240L	1 (3%)	0
Het: c.723G>C/p.(Glu241Asp), E241D	2 (5%)	0
Het: c.722A>G/p.(Glu241Gly), E241G	1 (3%)	0
Het: c.721G>A/p.(Glu241Lys), E241K	1 (3%)	0
Het: c.596A>G/p.(His199Arg), H199R	1 (3%)	1 (12%)
Het: c.690G>T/p.(Lys230Asn), K230N	1 (3%)	0
Het: c.725T>G/p.(Val242Gly), V242G	1 (3%)	0
Het: c.727G>A/p.(Asp243Asn), D243N	1 (3%)	1 (12%)
Het: c.855G>C/p.(Gln285His), Q285H	1 (3%)	0
**Inheritance**		
Autosomal dominant	23 (61%)	
*De novo*	5 (13%)	
Mosaic*	2 (5%)	
Unknown	8 (21%)	

Abbreviation: Het: heterozygous.

Data are presented as number (percentages). Amino acid substitutions are annotated according to HGVS. COPA (ENST00000241704.8 / NM_004371.4 / NP_004362.2).

* For F14.P1, the variant allele frequency (VAF) was 29% and 17% in blood and the kidney biopsy respectively; for F15.P1, the VAF was 10% in blood, 9% in nails, 12% in hair bulbs and 8% in buccal swab.

## Key Messages

### What is already known

- COPA syndrome is a rare monogenic type I interferonopathy.
- Known features of the disease are lung, joint and kidney disease.

### What this study adds

- Expansion of the known phenotype with skin, cardiac, digestive and hepatic involvement.
- Possibility of clinical non penetrance, *de novo* or somatic mutations.
- Morbidity and mortality driven by pulmonary and renal disease.
- Promising efficacy of JAK inhibition.

### How this study might affect research, practice or policy

- Sequence the *COPA* gene in the context of a concordant phenotype, even in the absence of a family history of disease.
- Perform a chest computed tomography scan, pulmonary function tests and a screen for proteinuria in any patient carrying a *COPA* mutation.
- JAK inhibition can be considered as a first-line treatment and anifrolumab may be a promising therapy to be trialled in COPA syndrome.
