# Supplementary material for: Insights from a novel monogenic autoinflammatory disease: overview of a multicentric European cohort of 38 patients with COPA syndrome
Source: Ann Rheum Dis. Author manuscript; Available in PMC 2026 Mar 1. (PMC7618534; doi:10.1016/j.ard.2025.09.013)
Supplement: Supplementary Material [file EMS211645-supplement-Supplementary_Material.docx]

**Supplementary Table**

**Supplementary Table S1. Detailed characteristics of the symptomatic patients in the cohort.**

| **Patient** | **Gender** | | **Mutation, inheritance** | **Age at report (years)** | **Age of onset (years)** | **Features at onset** | **Lung** | **Joint** | **Kidney** | **Skin** | **Hepatic** | **Digestive** | **Cardiac** | **Other** | **Auto-Ab** | **Discontinued treatment** | **Latest treatment** | **Previouspublication** |  |
| --- | --- | --- | --- | --- | --- | --- | --- | --- | --- | --- | --- | --- | --- | --- | --- | --- | --- | --- | --- |
| F1.P1 | F | | R233H, inherited | 18 | 2 | Lung | ILD and AH | - | - | - | - | - | - | - | ANA | Steroids, Cy, AZA, MMF, HCQ, anti-IL1, Ruxo | Bari | F2.P1 in (6) |  |
| F2.P1 | M | | R233H, inherited | 20 | 1 | Lung, fever, hepatitis | ILD and AH | Arthralgia | - | - | Cytolytic hepatitis | - | - | - | ANA, ANCA anti-MPO | Steroids, MMF, AZA, RTX | Ruxo | F3.P1 in (6), IV.2 in (12) |  |
| F2.P2, sister of F2.P1 | F | | R233H, inherited | 17 | NR | Skin | - | - | - | Vitiligo | - | - | - | - | NR | No treatment |  | F3.P2 in (6) |  |
| F2.P3, maternal cousin of F2.P1 | M | | R233H, inherited | Deceased (unknown) | NR | Lung | AH | - | - | - | - | - | - | - | NR | No treatment |  | F3.P4 in (6), III.1 in (12) |  |
| F2.P4, maternal cousin of F2.P1 | F | | R233H, inherited | 24 | 10 | NR | AH on BAL | Arthralgia | Lupus-like GN | - | - | - | - | - | Negative | Steroids, Cy, MMF, IVIG | Kidney Tx (18 y) | F3.P5 in (6), III.9 in (12) |  |
| F2.P5, maternal uncle of F2.P1 | M | | R233H, inherited | 60 | 50 | Kidney | - | - | Lupus-like membranous GN | - | - | - | - | - | NR | NR | NR | F3.P6 in (6), II.IV in (12) |  |
| F3.P1 | M | | R233H, inherited | Deceased (31) | 7 | Lung, joint | ILD | Deforming arthritis | - | Acrosyndrome | - | - | Cardiac hypertrophy | Nasal perforation, necrotizing sinusitis | ANA, ANCA, anti-RNP | HCQ | Lung Tx (28y), MMF, Tacro, steroids | F4.P1 in (6) |  |
| F4.P1 | F | | Q285H, de novo | 19 | 6 | Joint | - | Polyarticular JIA | - | - | - | - | - | - | anti-CCP / RF + | MTX | RTX | Patient in (10) |  |
| F5.P1 | F | | V242G, unknown | 30 | 8 | Joint | ILD | Destructive JIA, knee arthroplasty, Jaccoud-like | - | - | - | Chronic diarrhoea | Pulmonary hypertension | - | ANA, ANCA anti-MPO, anti-CCP | Steroids, NSAID, Salazopyrine, MTX, TCZ, IFX, ETC, Ciclo | Filgo | Patient in (22) |  |
| F6.P1 | F | | D243N, inherited | 18 | 2.5 | Joint | ILD | Arthralgia | - | Malar rash | HSMG | GERD | - | - | ANA, RF | Steroids, MMF, RTX, Bari | None | F1.P1 in (6) |  |
| F7.P1 | F | | W240R, inherited | 12 | 1 | Lung | ILD | - | - | - | - | - | - | - | ANA | No treatment | None | None |  |
| F7.P2, father of F7.P1 | M | | W240R, inherited | Deceased (35) | NR | Lung | ILD | - | - | - | - | - | - | - | ANA | NR | NR | None |  |
| F8.P1 | F | | R281W, inherited | 11 | 3 | Lung | ILD | Boutonniere deformity of 4^th^ finger | - | - | - | - | - | - | ANA | Steroids, MTX, MMF, ETC | Bari | None |  |
| F8.P2, sister of F8.P1 | F | | R281W, inherited | Deceased (23) | 10 | NR | ILD and AH | Polyarticular arthritis | - | Acral ulcers | - | - | Lymphocytic myocarditis | - | ANA | Steroids, Cy, MMF, ETC, RTX, TCZ, ABC | NR | None |  |
| F8.P3, brother of F8.P1 | M | | R281W, inherited | 30 | NR | NR | ILD | Arthralgia | - | - | - | - | - | - | NR | NR | NR | None |  |
| F9.P1 | F | | R281W, inherited | 5 | 2 | Joint | ILD | Wrist arthritis | - | - | - | - | - | - | ANA, RF | Steroids | Bari | Patient in (21) |  |
| F10.P1 | F | | R233H, inherited | 14 | 3 | Lung, joint | ILD | Destructive arthritis (wrist, hand, cervical spine) | - | - | - | - | - | - | ANA, RF | Steroids, NSAID, HCQ, MTX, MMF, ABC, RTX | Bari | Patient in (3) |  |
| F11.P1 | M | | W240R, inherited | 9 | 4 | Joint | - | Symmetrical polyarthritis | - | - | Cytolytic hepatitis under MTX | - | - | - | RF, anti-CCP | Steroids, MTX, ETC, Bari | Steroids, Tofa | None |  |
| F12.P1 | M | | R233H, inherited | 19 | 15 | Joint and skin | ILD | Joint arthritis | Pauci-immune ANCA-like GN | Livedo reticularis | - | - | - | - | ANA, ANCA anti-MPO, RF | Steroids, Cy, RTX, Ruxo | Kidney Tx (18 y), Bari, MMF, Tacro | None |  |
| F13.P1 | F | | H199R, inherited | 3 | 0 | Lung | ILD | - | - | - | - | - | - | - | NR | Steroids | Bari | Patient in (15) |  |
| F14.P1 | F | | R281W, mosaicism | 18 | 4 | Skin, kidney, gut | ILD | - | Pauci-immune ANCA-like GN | Purpura | - | Stercoral peritonitis | - | - | ANA, ANCA anti-MPO | Steroids, AZA, RTX | None | Patient 1 in (27) |  |
| F15.P1 | M | | R233H, mosaicism | 17 | 4 | Joint | ILD | Polyarticular arthritis | - | Psoriasis | - | - | - | Dyslexia | RF, anti-CCP | NSAIDs, MTX, ADM | Bari | Patient 2 in (27) |  |
| F16.P1 | F | | E241D, de novo | 23 | 0.3 | NR | ILD | Destructive polyarthritis | - | - | - | GERD, gastro-intestinal IgA vasculitis | Pulmonary hypertension | - | ANA, RF | Steroids, HCQ, Bari, Ruxo | RTX, NSAID | None |  |
| F17.P1 | M | | W240S, unknown | 31 | 14.5 | NR | ILD and AH | Non deforming polyarthritis | - | Malar rash | - | - | Mitral insufficiency | Myopia | ANA | Steroids, AZA | MMF, HCQ | None |  |
| F18.P1 | F | | W240L, de novo | 23 | 1.5 | NR | ILD and AH | Knee arthritis, scoliosis | Kidney insufficiency* | - | - | GERD | Pulmonary hypertension | Thyroiditis | ANA, ANCA anti-PR3, RF, anti-cardiolipin | MMF, MTX, Ciclo, TCZ, CAM, Ruxo, Bari, HCQ | Lung Tx (23 y), steroids, MMF, Tacro | None |  |
| F19.P1 | M | | R281W, inherited | 22 | 6 | NR | ILD | Arthralgia, scoliosis | - | - | Cytolytic hepatitis under MTX | - | Pulmonary hypertension | Retro-cerebellar cyst | ANA, ANCA anti-MPO and anti-PR3 | Steroids, MTX | HCQ, NSAID | None |  |
| F19.P2, mother of F19.P1 | F | | R281W, inherited | NR | NR | NR | ILD | Arthralgia | - | - | - | - | - | - | NR | NR | NR | None |  |
| F20.P1 | F | | R233H, de novo | 22 | 7.5 | NR | ILD | JIA | - | - | - | GERD | Cardiac insufficiency | - | ANA, ANCA anti-MPO, RF, anti-centromere | Steroids, ETC, ADM, LEF, TCZ, GOM, MTX, Bari | Upadacitinib | None |  |
| F21.P1 | F | | R281W, inherited | 14 | 5.5 | NR | ILD and AH | Arthralgia | Kidney insufficiency | - | - | - | - | - | ANA, anti-DNA, ANCA | NSAID | Bari | None |  |
| F21.P2, father of F21.P1 | M | | R281W, unknown | NR | NR | NR | ILD | Polyarthritis | - | Skin disease without precision | - | - | - | - | ANA | NR | NR | None |  |
| F22.P1 | M | | W240S, unknown | 5 | 0.5 | NR | ILD and AH | - | - | - | - | - | - | - | ANA | Steroids | Bari, NSAID | None |  |
| F23.1 | M | | W240R, inherited | 8 | 3 | NR | ILD | - | - | - | - | - | - | - | NR | Steroids | None | None |  |
| F24.1 | M | | K230N, unknown | 19 | 0.5 | NR | ILD | JIA | - | - | - | - | - | - | ANA | Tofa,Ruxo | Steroids, ETC | None |  |
| F25.1 | F | | R233H, unknown | 9 | 1 | Lung, skin, joint | ILD | Arthralgia | - | Non-specific rash, aphthae | Cytolytic hepatitis | Diarrhoea | - | - | ANA, anti-SLA | NSAID, HCQ | Ruxo | None |  |
| F26.1 | F | | R233H, unknown | 14 | 2 | Joint and skin | AH | Ankle arthritis | Pauci-immune ANCA-like GN | Purpura | - | - | - | - | ANCA anti-MPO | Steroids, MMF, HCQ | Bari | None |  |
| F27.1 | F | | E241D, inherited | 58 | 14 | Joint | ILD | Polyarthritis and deforming arthritis | - | Transient chilblains and panniculitis (30 y) | - | - | - | - | ANA, anti-RNP, ANCA anti-MPO, RF, anti-CPP | Steroids, MMF, MTX, LEF, HCQ, AZA, TCZ, RTX | Bari, steroids | None |  |
| F28.1 | F | | E241G, de novo | 4 | 2 | Lung | ILD and AH | - | - | - | - | - | - | - | ANCA anti-MPO | Steroids | Bari | None |  |
| F29.1 | M | | E241K, unknown | 0.5 | 0.08 | Lung | ILD | - | - | - | - | - | - | - | ANCA anti-MPO | None | Bari | None |  |
|  | | Abbreviations: ABC: abatacept; ADM: adalimumab; AH: alveolar haemorrhage; ANA: antinuclear antibodies; ANCA: anti-neutrophil cytoplasmic antibodies; AZA: azathioprine; Bari: baricitinib; CAM: canakinumab; Ciclo: cyclosporine; Cy: cyclophosphamide; ETC: etanercept; F: female; Filgo: Filgotinib; GERD: gastroesophageal reflux disease; GN: glomerulonephritis; GOM: golimumab; HSMG: hepatosplenomegaly; HCQ: hydroxychloroquine; IFX: infliximab; ILD: interstitial lung disease; IVIG: intravenous immunoglobulins; JIA: juvenal idiopathic arthritis; LEF: leflunomide; M: male; MMF: mycophenolate mofetil, MTX: methotrexate, NR: not recorded; NSAID: non-steroidal anti-inflammatory drug; RTX: rituximab; Ruxo: ruxolitinib; Tacro: tacrolimus; TCZ: tocilizumab; Tofa: tofacitinib; Tx: transplantation; y: years.  *Occurred before lung transplantation | | | | | | | | | | | | | | | | | |

**Supplementary Table S2. Characteristics of lung involvement in the COPA cohort**

| Lung involvement | n=34 |
| --- | --- |
| *ILD* | 31 (91%) |
| *AH* | 11 (32%) |
| *ILD and AH* | 8 (24%) |
| *Fibrosis* | 14 (41%) |
| Clinical features | n=23 |
| *Cough* | 21 (91%) |
| *Dyspnoea* | 14 (61%) |
| *Tachypnoea* | 10 (43%) |
| *Nail clubbing* | 10 (43%) |
| *Haemoptysis* | 2 (9%) |
| *Asymptomatic lung disease* | 1 (3%) |
| CT scan abnormalities | n=25 |
| *Fibrosis* | 12 (48%) |
| *Ground glass opacities* | 22 (88%) |
| *Cysts* | 15 (60%) |
| *Honeycombing* | 6 (24%) |
| *Micronodules* | 5 (20%) |
| *Septal thickening* | 10 (40%) |
| *Hilar lymphadenopathy* | 5 (20%) |
| Pulmonary function tests | n=19 |
| *Normal* | 5 (26%) |
| *Restriction* | 13 (87%) |
| *Obstruction* | 1 (5%) |
| *Mixed pattern* | 4 (21%) |
| BAL analysis | n=16 |
| *Normal* | 1 (6%) |
| *Lymphocytic alveolitis* | 8 (50%) |
| *AH* | 6 (38%) |
| *Neutrophilic alveolitis* | 2 (13%) |
| *Lipid-laden macrophages* | 2 (13%) |
| Lung biopsy | n=10 |
| *Fibrosis* | 5 (50%) |
| *Follicular hyperplasia* | 10 (100%) |
| *Inflammatory lymphocytic infiltrates* | 6 (60%) |
| *Follicular bronchiolitis* | 5 (50%) |
| *Cholesterol pneumonitis* | 2 (20%) |
| *Focal organizing pneumoniae* | 1 (10%) |
| *Cellular non-specific interstitial pneumonia* | 1 (10%) |
| *Fibrous pleuritis* | 1 (10%) |
| *Alveolar simplification* | 1 (10%) |
| Supportive therapy during the course of the disease | 6 (18%) |
| *Oxygen therapy* | 6 (18%) |
| *Non-invasive ventilation*  *Invasive ventilation* | 3 (9%)  1 (3%) |
| *Lung transplantation* | 2 (6%) |
| *Awaiting lung transplantation* | 1 (3%) |

Data are presented as number (percentages).

Abbreviations: AH: alveolar haemorrhage, BAL: bronchoalveolar lavage; CT: computed tomography; ILD: interstitial lung disease.

**Supplementary Table S3. Treatments received by patients in the cohort during the course of the disease.**

| Corticosteroids | 24 (63%) |
| --- | --- |
| NSAIDs | 6 (16%) |
| Intravenous immunoglobulins | 1 (3%) |
| Hydroxychloroquine | 8 (21%) |
| DMARDs | 21 (55%) |
| *Cyclophosphamide* | 4 (11%) |
| *Azathioprine* | 6 (17%) |
| *Mycophenolate mofetil* | 15 (39%) |
| *Methotrexate* | 8 (21%) |
| *Ciclosporin* | 2 (5%) |
| *Tacrolimus* | 3 (8%) |
| *Sirolimus* | 1 (3%) |
| *Leflunomide* | 2 (5%) |
| Biotherapy | 17 (47%) |
| *Rituximab (anti-CD20 mAb)* | 9 (25%) |
| *Tocilizumab (anti-IL6)* | 5 (14%) |
| *Anti-IL1 therapy* | 2 (6%) |
| *Anti-TNF* | 10 (28%) |
| *Abatacept* | 2 (5%) |
| JAK inhibitors | 22 (58%) |
| *Ruxolitinib (JAK1/2)* | 6 (16%) |
| *Baricitinib (JAK1/2)* | 17 (45%) |
| *Tofacitinib (JAK1/3)* | 3 (8%) |
| *Upadacitinib (JAK1)* | 1 (3%) |
| *Filgotinib (JAK1)* | 1 (3%) |
| Kidney transplant | 2 (5%) |
| Lung transplant | 2* (5%) |
| Abbreviations: DMARDs: disease-modifying antirheumatic drugs; JAK: Janus kinase; IL: interleukin; mAb: monoclonal antibody; NSAIDs: non-steroidal anti-inflammatory drugs; TNF: Tumour Necrosis Factor.  Data are presented as number (percentages). | |

*One patient died of an out-of-hospital cardiac arrest 4 years post lung transplantation.

**Supplementary TableS4. Immunological features of the symptomatic patients.**

| Immunological features |  |
| --- | --- |
| **Auto-antibodies (n = 31 tested)** | 30(97%) |
| ANA | 24 (77%) |
| *Anti-DNA* | 1 (3%) |
| *Other* | Anti-RNP (n=2), anti-centromere (n=1), anti-Scl70 (n=1) |
| ANCA | 14(45%) |
| *Anti MPO* | 9(29%) |
| *Anti PR3* | 2 (6%) |
| *Anti-MPO and anti-PR3* | 2 (6%) |
| *Without specificity* | 1 (3%) |
| Anti-CCP | 6(19%) |
| Rheumatoid factor | 12 (39%) |
| Other | Anti-cardiolipin (n=1), anti-SLA (n=1) |
| **Positive interferon signature (n=20 tested)** | 20 (100%) |
| **High ESR (n=11 tested)** | 9 (82%) |
| **Elevated CRP (n=19 tested)** | 7 (37%) |
| **ESR / CRP dissociation (n=11 tested)** | 6 (55%) |
| **Hypergammaglobulinemia (n=13 tested)** | 7 (54%) |
| **Lymphocyte immunophenotyping (n=7 tested)** | NK lymphopenia, decrease of naïve T CD4+ with increase percentage of naive T CD8+, with a decrease in central memory, memory effectors and TEMRA (n=1), isolated NK lymphopenia (n=1), moderate T CD4+ and CD8 + lymphopenia (n=2), excess of naïve T CD4+ (n=1), decrease of memory T CD8+with increase of naïve T CD8+ (n=1), normal (n=1). |
| Abbreviations: ANA: antinuclear antibodies, ANCA: anti-neutrophil cytoplasmic antibody, CCP: anti-citrullinated protein; CRP: C-reactive protein; ESR: erythrocyte sedimentation rate, TEMRA:effector memory cells re-expressing CD45RA. | |

**Supplementary Table S5. Inflammatory and immunological features of the symptomatic patients in the cohort.**

| **Patient** | **IFN signature** | **Positive CRP** | **Positive ESR** | **Immunoglobulins level** | **Lymphocyte immunophenotyping** |
| --- | --- | --- | --- | --- | --- |
| F1.P1 | Positive | Yes | NR | Normal | NK lymphopenia, decrease of naïve T CD4+ with increase percentage of naive T CD8+, with a decrease in central memory, memory effectors and TEMRA |
| F2.P1 | Positive | Yes | NR | NR | Moderate T CD4+ and CD8 + lymphopenia |
| F2.P2 | NR | NR | NR | NR | NR |
| F2.P3 | NR | NR | NR | NR | NR |
| F2.P4 | Positive | NR | NR | NR | NR |
| F2.P5 | Positive | No | Yes | NR | NR |
| F3.P1 | Positive | NR | NR | NR | NR |
| F4.P1 | Positive | NR | NR | NR | NR |
| F5.P1 | Positive | No | Yes | Hypergammaglobulinemia | Excess of naïve T CD4+ |
| F6.P1 | Positive | NR | NR | NR | NR |
| F7.P1 | Positive | No | Yes | Hypergammaglobulinemia | Normal |
| F7.P2 | NR | NR | NR | NR | NR |
| F8.P1 | Positive | No | Yes | Hypergammaglobulinemia | NR |
| F8.P2 | NR | NR | NR | NR | NR |
| F8.P3 | NR | NR | NR | NR | NR |
| F9.P1 | Positive | No | Yes | Hypergammaglobulinemia | NR |
| F10.P1 | NR | No | Yes | NR | NR |
| F11.P1 | Positive | NR | NR | NR | NR |
| F12.P1 | Positive | No | NR | NR | NR |
| F13.P1 | Positive | NR | NR | NR | NR |
| F14.P1 | Positive | NR | NR | Post-rituximab hypogammaglobulinemia | Isolated NK lymphopenia |
| F15.P1 | Positive | NR | NR | NR | NR |
| F16.P1 | NR | Yes | No | NR | NR |
| F17.P1 | NR | Yes | NR | NR | NR |
| F18.P1 | NR | Yes | No | NR | NR |
| F19.P1 | NR | No | NR | NR | NR |
| F19.P2 | NR | NR | NR | NR | NR |
| F20.P1 | NR | No | NR | NR | NR |
| F21.P1 | NR | No | Yes | Normal | NR |
| F21.P2 | NR | NR | NR | NR | NR |
| F22.P1 | NR | NR | NR | Normal | NR |
| F23.1 | NR | NR | NR | Normal | NR |
| F24.1 | NR | Yes | NR | Hypergammaglobulinemia | NR |
| F25.1 | Positive | NR | Yes | Hypergammaglobulinemia | NR |
| F26.1 | Positive | NR | NR | NR | Moderate T CD4+ and CD8 + lymphopenia |
| F27.1 | Positive | Yes | Yes | Normal | NR |
| F28.1 | NR | No | NR | Hypergammaglobulinemia | NR |
| F29.1 | Positive | No | NR | NR | decrease of memory T CD8+ with increase of naïve T CD8+ |

Abbreviations: CRP: C-reactive protein; ESR: erythrocyte sedimentation rate, NR: not recorded; TEMRA:effector memory cells re-expressing CD45RA.

**Supplementary Table S6. Response to JAK inhibitors in the cohort**

|  | **Indication of JAKi** | **Age at initiation (y)** | **JAKi** | **Dosage at initiation** | **Dosage Max** | **Last dosage** | **Concomitant IS** | **Efficacy** | **Concomitant IS cessation** | **Cessation of JAKi** | **Follow-up (months)** | **Side effects** |
| --- | --- | --- | --- | --- | --- | --- | --- | --- | --- | --- | --- | --- |
| **F1.P1** | Severe AH with multiple life-threatening decompensations | 11 | Ruxo | 30mg/day | 50mg/day | 50mg/day | Steroids  Sirolimus | Partial remission with no severe DAH flares. | Steroids  Sirolimus (BK viremia) | Yes, partial efficacy | 25 | Gain of weight |
|  | Partial efficacy of Ruxolitinib | 14 | Bari | 8mg/day | 12mg/day | 4mg/day | None | Remission | No | No | 48 | BK viremia; Meningococcalpneumonia |
| **F2.P1** | Recurrent AH | 16 | Ruxolitinib | 30mg/day | 40mg/day | 40mg/day | None | Clinical remission | Yes | No | 46 | Alopecia |
| **F5.P1** | Polyarthritis | 29 | Filgo | 100mg/day | 100mg/day | 100mg/day | Steroids | Remission of joint disease  Lung fibrosis stable  Progression of PAH | Steroids | No | 13 | None |
| **F6.P1** | Gradual lung function deterioration | 13 | Bari | 4mg/day | 8mg/day | 8mg/day | MMF  RTX  Steroids | Stabilisation of lung function | MMF | Yes, non-compliance | 58 | Abdominal pain |
| **F8.P1** | Pulmonary involvement | 5 | Bari | 4mg/day | 6mg/day | 6mg/day | No | Stabilisation | No | No | 72 | None |
| **F9.P1** | Pulmonary involvement | 2.5 | Bari | 4mg/day | 4mg/day | 4mg/day | Steroids | Clinical improvement | Steroids | No | 24 | None |
| **F10.P1** | Lack of response to previous IS | 12 | Bari | 4mg/day | 6mg/day | 6mg/day | None | Complete response (one post viral joint flare requiring dose escalation) | Yes | No | 48, then lost to follow-up | None |
| **F11.P1** | Polyarthritis | 6 | Bari | 6mg/day | 8mg/day | 8mg/day | Steroids | Initial remission then loss of efficiency | No | Yes, loss of efficacy | 33 | Non |
|  | Loss of efficacy of bari | 8 yr | Tofa | 8mg/day | 12mg/day | 12mg/day | Steroids | Low efficiency | No | No | 13 | None |
| **F12.P1** | Polyarthritis | 16 | Ruxo | 5mg/day | 5mg/day | 5mg/day | RTX, steroids | Partial | No | Yes, kidney transplant | 4 | None |
|  | Switch post kidney transplantation | 17 | Bari | 4mg/day | 4mg/day | 4mg/day | MMF, Tacro | Partial | No | No | 17 | None |
| **F13.P1** | ILD | 1.5 | Bari | 3,6mg/day | 6 mg/day | 6 mg/day | None | Improvement | No | No | 24 | None |
| **F15.P1** | ILD | 15 | Bari | 4mg/day | 4mg/day | 4mg/day | Adalimumab | Joint and walking test improvement, stabilisation of PFT | Decrease of adalimumab rhythm of infusion | No | 24 | None |
| **F16.P1** | ILD and arthralgia | 21 | Bari | 12mg/day | 12mg/day | 12mg/day | Naproxen | Inefficacy on joint disease | No | Yes, for inefficiency | 10 | None |
|  | Inefficacy of bari | 21 | Ruxo | 30mg/day | 30mg/day | 30mg/day | Naproxen | Worsening of joint disease | No | Yes, for inefficiency | 2 | None |
| **F18.P1** | ILD and polyarthritis | 19 | Bari | 4mg/day | 8mg/day | 8mg/day | Steroids,  HCQ, MTX | Progression of ILD requiring lung Tx | Yes,switch for MMF, Tacro and steroids (lung Tx) | Yes, after lung transplant | 48 | None |
| **F20.P1** | Arthralgia |  | Bari | 4mg/day | 8mg/day | 8mg/day | Steroids, MTX, Naproxen | Good response but loss of efficiency | No | Yes, loss of efficiency | 66 | None |
|  |  |  | Upada | 15mg/day | 30mg/day | 30mg/day | MTX | Satisfying | Yes | No | 24 | None |
| **F21.P1** | ILD and arthralgia | 12.5 | Bari | 8mg/day | 12mg/day | 8mg/day | Naproxen | Stable | Yes, Naproxen | No | 22 | Gastro-intestinal complaints requiring tapering of dosage |
| **F22.P2** | ILD | 1.5 | Bari | 2mg/day | 6mg/ day | 4mg/ day | Naproxen | Stable | No | No | 49 | Leucopenia, elevation of liver enzymes |
| **F24.1** | ILD | 15.5 | Tofa | 10mg/day | 10mg/day | 10mg/day | Steroids,  Etanercept | Reduction in pulmonary symptoms but degradation of CT scan and PFT | No | Yes,for inefficiency | 13 | None |
|  |  | 17 | Ruxo | 20mg | 20mg | 20mg | Steroids, ADM | Deterioration of CT scan and PFT | No | No | 26 | None |
| **F25.P1** | ILD and polyarthritis | 8 | Ruxo | 10mg/day | 10mg/day | 10mg/day | No | Improvement | No | No | 7 | None |
| **F26.P1** | Skin and arthritis | 13 | Bari | 2mg/day | 4mg/day | 4mg/day | Steroids, MMF, HCQ | Remission | Yes, steroids and HCQ stopped | No | 3 | None |
| **F27.P1** | Progression of lung fibrosis with dyspnoea and chronic cough, arthralgias | 57 | Bari | 2mg/day | 4 mg/day | 4 mg/day | Steroids and MMF | Good efficiency on respiratory symptoms but severe polyarthritis flare under treatment | Yes; MMF stopped and  Prednisone decreased | Yes, severe polyarthritis needing RTX therapy | 5 | Paradoxical polyarthritis flare? |
| **F28.P1** | AH | 3.25 | Bari | 4mg/day | 4mg/day | 4mg/day | Steroids | Improvement | Yes | No | 12 | None |
| **F29.P1** | Lung disease | 0.5 | Bari | 2mg/day | 2mg/day | 2mg/day | No | Just started | No | No | 0 | None |
| Abbreviations: ADM: adalimumab; AH: alveolar haemorrhage; Bari: baricitinib; Filgo: filgotinib; HCQ: hydroxychloroquine; MMF: mycophenolate mofetil; MTX: methotrexate; PAH: pulmonary arterial hypertension; PFT: pulmonary function test; RTX: rituximab; Ruxo: ruxolitinib; Tacro: tacrolimus; Tofa: tofacitinib; Tx: transplantation; Upada: upadacitinib | | | | | | | | | | | | |
